# Advancing the Manufacture of Metal Anodes for Metal Batteries

**DOI:** 10.1021/accountsmr.3c00231

**Published:** 2024-01-26

**Authors:** Pan He, Yupei Han, Yang Xu

**Affiliations:** Department of Chemistry, University College London, London WC1H 0AJ, U.K.

## 1 Introduction

The rapid research progression in metal batteries (MBs) highlights the importance of metal anodes, the most energy-dense choice among all anodes. Metal anodes involve alkali metals (Li, Na, and K)^1^ and multivalent metals (Mg, Ca, Zn, and Al),^2^ and they are usually utilized in the form of metal foils. However, the practical application of metal anodes is accompanied by notorious challenges such as safety risks induced by metal dendrite growth, low Coulombic efficiency (CE) caused by parasitic reactions and “dead metal”, unstable solid electrolyte interphase (SEI), and low utilization of metal anodes due to their excessive thicknesses.^3^

The intrinsic properties of metal anodes, including geometric structure, surface roughness, crystal orientation, grain size, defect, etc., are closely related to the manufacturing process. These properties play a decisive role in determining the electrochemical performance of metal anodes. In addition, the storage and processing atmosphere (e.g., compositions of volatile solvent gases and oxygen/moisture level in a glovebox) affects the surface species of metal anodes.^4^ As a result, it is crucial to clarify the dominating factors and optimize the repeatability, generality, and scalability of the manufacturing process from raw metal materials to metal anodes for MBs.

In this Viewpoint, we divide the manufacture of metal anodes into three steps: pretreatment, processing, and post-treatment. We start with the discussion on the fundamental but often overlooked pretreatment step, then compare various processing methods and highlight the imperfections of metal anodes that may form during the processing step. Finally, we discuss post-treatment strategies to effectively optimize the electrochemical plating behavior of metal anodes. To conclude our discussion, we propose a sequential and scalable solution for metal anode manufacture, hoping to spark further research innovation in the exciting area of MBs.

## 2 Pretreatment and Processing

**Figure 1:**
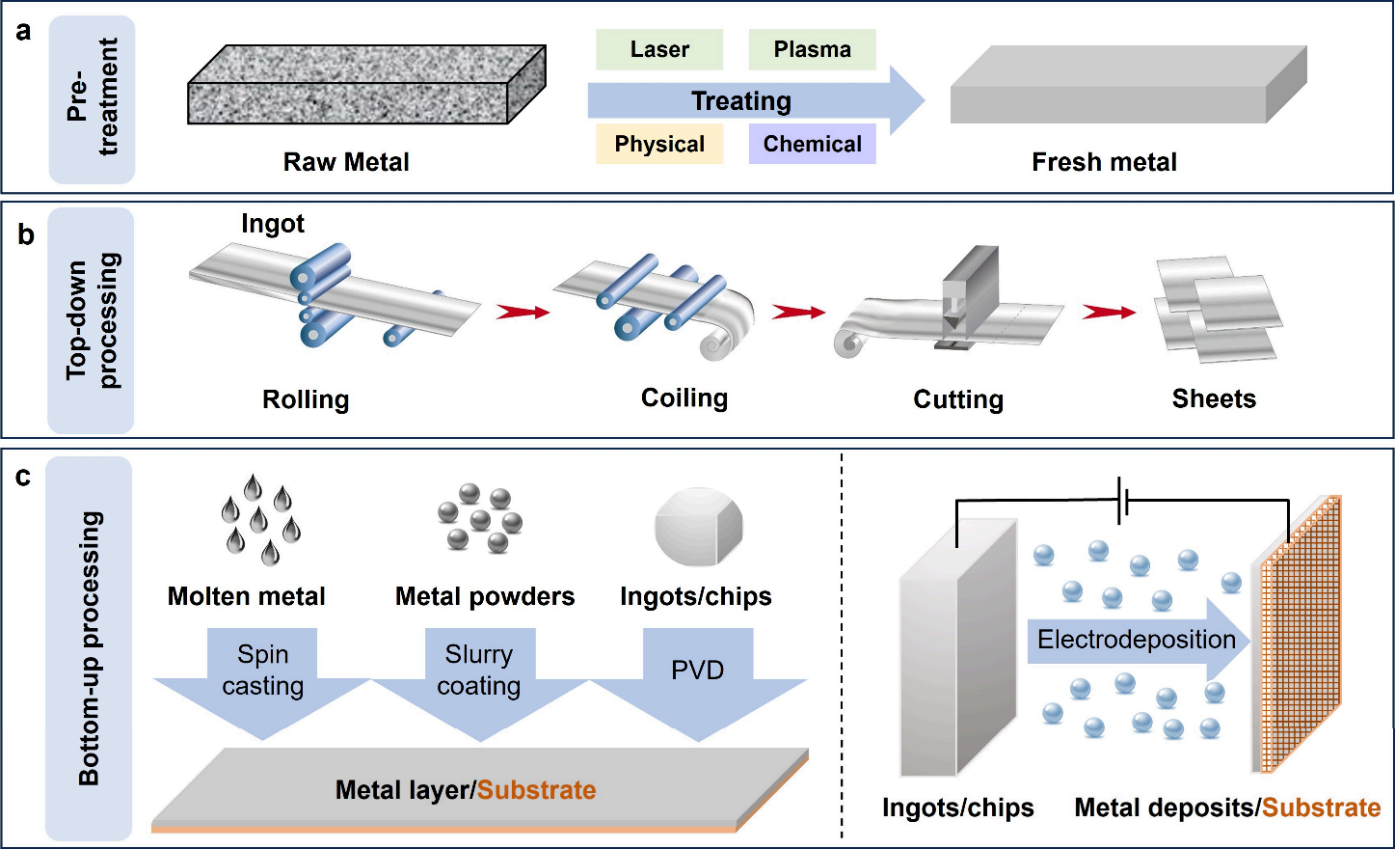
Illustrations of pretreatment methods and processing strategies for metal anodes. (a) The diagram shows typical pretreatment methods for raw metals, including physical and chemical, as well as mixed/advanced approaches, such as laser and plasma processes. All the pretreatment methods can produce a fresh surface of the foil. (b) The diagram shows a typical top-down process to produce metal anodes, which involves rolling, coiling, and cutting processes.^9^ Reproduced with permission from ref (9). Copyright 2021 American Chemical Society. (c) The diagram shows a typical bottom-up process to prepare metal anodes. Metal anodes can be fabricated using various physical methods, such as spin-casting of molten metals, slurry coating of metal powders, and PVD for ingots/chips. In addition, metal anodes can be electrodeposited onto a 3D current collector.

Highly reactive metals such as Li, Na, K, and Ca need to be stored and processed in a glovebox filled with a high-purity (>99.99%) inert gas (Ar for Li and Ca; Ar or N_2_ for Na and K). In addition, Na and K are immersed in an inert medium, such as hexane, kerosene, etc., for proper storage. Nevertheless, chemical reactions with surroundings are unavoidable for these metals, leaving a thin layer of metal oxides, hydroxides, or carbonates on the surface.^5^ Regarding less reactive metals of Mg, Zn, and Al, they can be handled in an ambient atmosphere, but are unavoidable to form undesired dense passivation layers. Therefore, as shown in Figure 1a, essential pretreatment of metal foils to obtain a clean surface is one of the most direct approaches to unlock the full potential of metal anodes. For example, a quick physical polishing of a Li foil with a silicon carbide paper in high-purity acetone for several seconds can remove impurities from the surface.^5^ A similar effect is observed from a chemical etching procedure for Mg foil.^6^ Other well-established surface cleaning techniques for alloys (e.g., stainless steel) and silicon wafers, such as laser polishing, chemical polishing, and plasma cleaning, have potential in pretreating metal foils.^7,8^ Note that all the pretreatment methods mentioned here have only been proven effective in small units/batches of metals with manual-handling in a laboratory setting and thus, making these methods automated, cost-effective, and scalable is particularly encouraged. Besides, one of the most overlooked but noteworthy issues is that all the metal anodes are not in absolute purity. The exact compositions and properties of impurities largely remain unknown, and their potential effects on the electrochemical performance of the anodes have yet to receive sufficient research attention, not to mention the understanding of the effects, which needs in-depth investigations.

Processing is the most important part of metal anode manufacture, as the inherent properties of metal foils are intricately linked to processing methods. Starting from metal ingots, a processing method based on a top-down approach typically involves a rolling process to achieve a desirable thickness, a coiling step to form rolls for easy storage and transportation, and a cutting step to obtain specific shapes and sizes (Figure 1b).^9^ The processing of Mg, Zn, and Al metals that are less reactive share the same top-down approach as Ti and stainless steel. An additional inert atmosphere is necessary for air-sensitive Li, Na, K, and Ca metal foils to avoid undesired side reactions and potential safety hazards, which not only increases the processing cost but also poses challenges to manufacture equipment and scalability. Thick metal anodes result in low utilization of the metal and thus decrease their overall energy density, while thin metal anodes are difficult to achieve processing feasibility; a balance needs to be reached. For example, a Li foil produced by the state-of-the-art top-down process can be as thin as 20 μm (∼4.2 mA h cm^–2^), which has just enough areal capacity to meet practical requirements, so the scalability of the process still needs further extension.^10^ Such a thin Li foil is prone to deformation or breakage and thus is mounted on a copper current collector to form a metal-current collector (MCC) composite.

Alternatively, processing methods based on a bottom-up approach are facile to produce a thin metal anode with a desirable thickness and/or an MCC composite (Figure 1c). Benefiting from the relatively low melting points (Li: 180.5 °C, Na: 97.8 °C, K: 63.5 °C, Zn: 419.5 °C, Mg: 650 °C, Al: 660.3 °C),^11^ these metals can ideally be processed into controlled geometries, such as thin films and MCC composites, through spin-casting of molten metals or slurry coating of metal powders. Particularly, three-dimensional (3D) porous conductive structures are widely achieved by casting/infusing molten metals into 3D porous skeletons (e.g., Cu foam, Ni foam, graphene, carbon nanotubes).^1,2^ Furthermore, the well-established physical vapor deposition (PVD) technique can be used to process metals at acceptable operating temperatures (Li: 400–600 °C, Na: 200–300 °C, K: 200–400 °C, Zn: 400–500 °C, Mg: 400–600 °C, Al: 600–700 °C).^11^ Note that the three bottom-up approaches, i.e., spin-casting, slurry coating, and PVD, need to be operated in a well-controlled atmosphere (e.g., inert and/or low pressure), which adds production cost. Compared to physical methods, electrodeposition can provide better conformality and uniformity of metal anodes, particularly in complex geometry structures, such as electrodeposition of a metal anode film onto a 3D current collector.

## 3 Imperfections Formed during Processing

**Figure 2:**
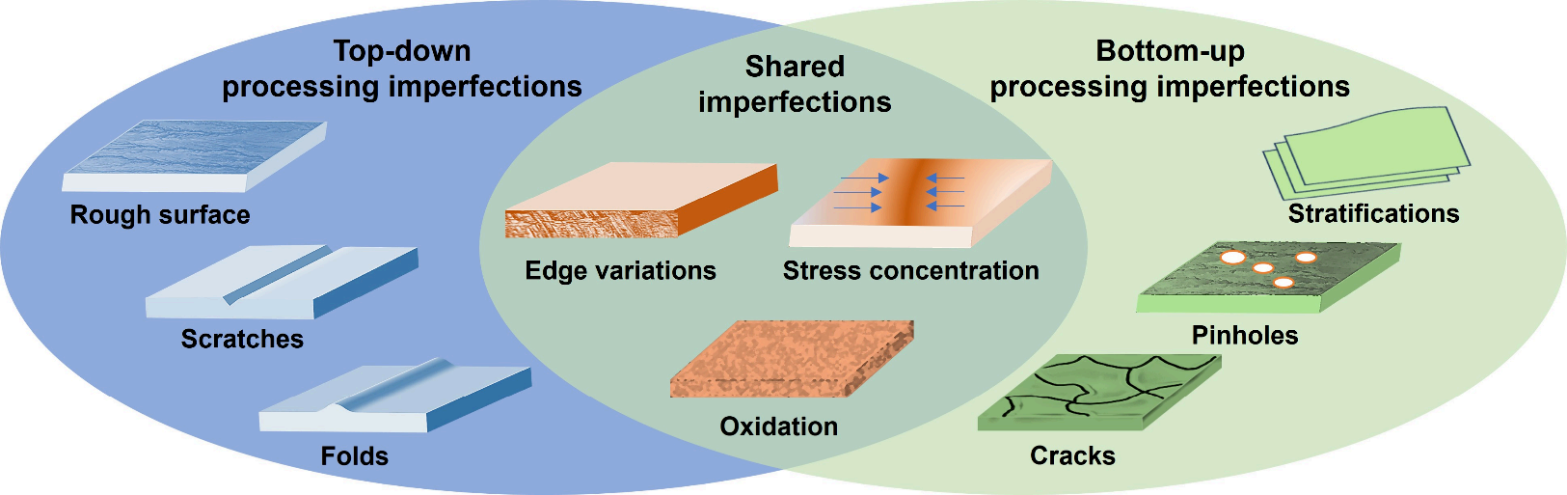
Illustrations of the imperfections introduced during the processing of metal anodes. The left part of the illustrations shows surface roughness, scratches, and folds introduced during a top-down pressing and rolling process; the right part of the illustrations shows pinholes, cracks, and stratifications introduced during a bottom-up process; the overlapping part of the illustrations shows the imperfections shared between the two processes, including oxidation, residual stress, and edge variations.

Imperfections can form during the metal processing step and have adverse effects on the electrochemical performance of metal anodes. Taking an example of the top-down rolling-pressing approach, different types of defects can be introduced at different processing steps (Figure 2).^9^ Irregular grains and/or textures can form on metal surfaces during repetitive rolling-pressing processes, resulting in an increased surface roughness and inhomogeneity. Scratches or creases are commonly formed during almost all processing and handling steps, depending on the softness of metals (Vickers hardness scale, Li: 5, Na: 0.3, K: 0.4, Zn: 40–50, Mg: 45–55, Al: 15, Ca: 15).^11^

Similarly, imperfections can be created during bottom-up processing, and this has yet to receive research attention. For example, defects such as shrinkage pinholes, porosity, hot tears, and cracks may form in a metal foil during a spin-casting process.^12^ As for the slurry coating approach, the poor dispersity of metal particles can lead to an inhomogeneous coating, and cracking and delamination may occur during the drying or curing process after coating. Stratification is often found in metal films produced by PVD. Regarding electrochemical deposition, incomplete coverage, inclusion of byproducts, and undesired morphologies are likely to be an issue leading to the performance degradation of metal anodes.

Furthermore, top-down and bottom-up processing approaches may result in common imperfections, such as surface oxidation, edge variations, and stress concentration, which often are not noticed. Real-time detection and monitoring of surface oxidation of metals are challenging. Edges may not be processed in the same way as surfaces, resulting in the properties deviating from surfaces. Particularly, a cutting step exposes fresh metal edges that are quite different from the surface. Moreover, in some cases, there is an uneven distribution of residual stress on the surface of metal anodes, but the correlation between the residual stress and metal plating remains unknown. Insights into the ramifications of the discussed imperfections of metal anodes can improve the processing approaches and more importantly, guide the following post-treatment step.

## 4 Post-treatment

**Figure 3:**
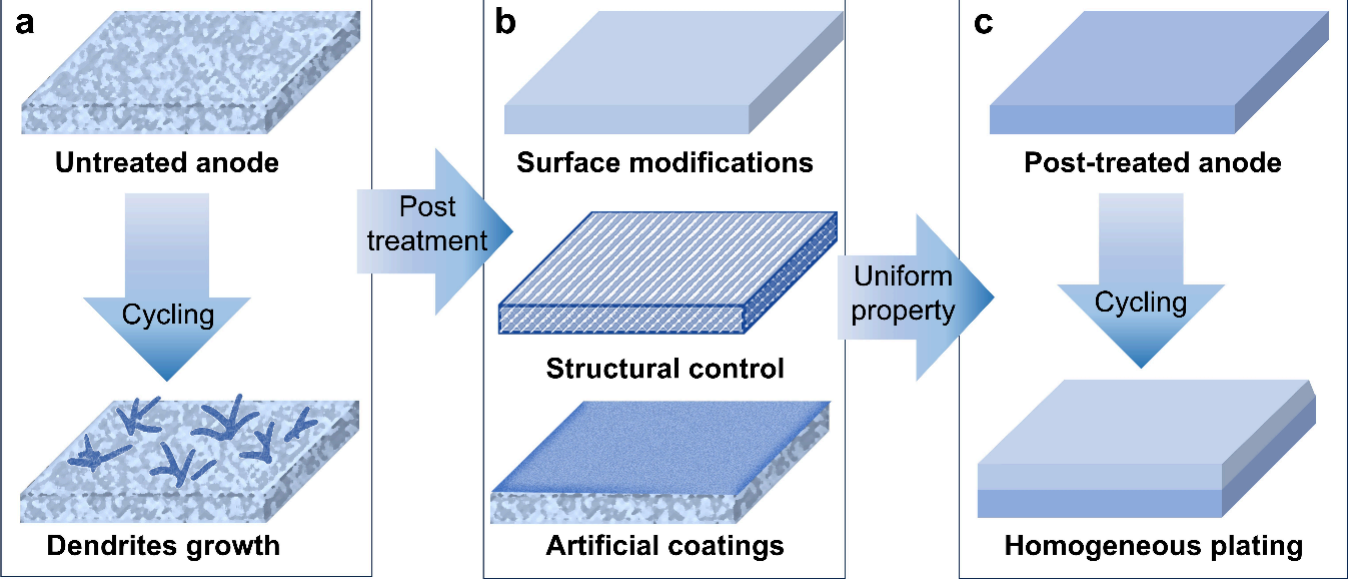
Plating behaviors of untreated and treated anodes. (a) Imperfections of untreated anodes trigger dendrite growth during electrochemical cycling. (b) Three typical post-treatment strategies include surface modifications, structural control, and artificial coatings can improve the uniformity of the anode, (c) thereby promoting uniform electrodeposition.

Commercially available metal foils/electrodes are typically manufactured using one or more of the processes discussed in the previous sections before they are used in a laboratory setting as “untreated metal anodes” that contain substantial nonuniformities in the bulk and a multitude of processing imperfections on the surface. Imperfect sites (e.g., scratches, protrusions, microscopic roughness, grain boundary, impurities) can alter the metal anode–electrolyte interface in a MB. For instance, the sites, acting as “hot spots”, induce an intensified local electric field that attracts ions and causes locally concentrated ion distribution during plating.^13^ Once electrodeposition is activated at the “hot spots” where metal ion concentration hits the threshold, uneven metal plating is initiated and promoted by the “tip effect”, leading to the formation and growth of dendrites (Figure 3a).^1,2^

Post-treatment on bulk metal or metal surfaces can minimize or even eliminate the negative effect of imperfections. As shown in Figure 3b, we summarize post-treatment into three strategies: surface modifications, structural control, and artificial coatings. Surface modifications work similarly to the pretreatment process. It involves a washing step to remove lubricant residuals from rolling, debris from cutting, and impurities from slurries or agents. Further elimination of the passivation layer and imperfections from the surface is achieved by polishing or etching. For instance, the chemical etching of a Li metal foil using naphthalene and physical polishing of a Zn metal foil with sandpaper to remove the native film from the surface, rendering significantly improved electrochemical performance of the anodes.^9,14^ The removal of a surface passivation layer lowers the interfacial energy between the metal surface and electrolyte, thereby facilitating the formation of a stable SEI layer and decreasing the ion diffusion barrier. Several positive results of surface modifications have been seen in the literature, which urges in-depth investigations into the correlation between the surface properties of the metal anode and the uniformity of metal plating.

Besides surface modifications to remove surface imperfections, designing a hierarchical structure on the surface of a metal anode is proven effective in regulating metal nucleation and deposition. Electrodeposition of a metal anode is largely affected by the surface energy difference originated from the lattice mismatch between the growing film and the substrate,^15^ and the effect can be minimized by exposing specific crystallographic planes on the metal anode surface. Particularly, a perfect homoepitaxial growth of Zn was achieved on an ideal single-crystal (002) plane of a Zn anode.^16^ Generally speaking, it is believed that a similar epitaxial electrodeposition process can be achieved for other metals (Li, Na, K, Mg, Al, and Ca) with a desirable exposed crystallographic plane or when applying a substrate with a lattice mismatch of <25%.^17^ However, it is still incredibly challenging to manufacture metal anodes with exposing a particular crystallographic plane at a large scale, not to mention retaining the plane during long-term cycling as the lattice mismatch to the growing metal film can gradually increase. Besides the epitaxial growth strategy, surface anchoring reactive seeds that are soluble (Au, Zn, Ag) in or electrochemically reactive (Sn, Si) to the metal anode can promote uniform nucleation.^18^ The challenge here is how to keep the seeds stable and active during repetitive metal deposition/dissolution. Furthermore, surface patterning on metal anodes (e.g., Li, Na, K, Zn) is another strategy to hinder dendrite growth. During the Li plating and stripping processes, liquid-like and/or granular-featured Li metal was observed to fill in and drain off reversibly from surface patterned holes that remain unchanged.^19^ Similarly, the uniform distribution of microgrooves can regulate an averaged Zn^2+^ allocation flux, thereby realizing stable Zn electrodeposition.^20^ Note that surface patterning needs to be facile and scalable as well as compatible with metal manufacturing processes, but it is unclear how the parameters of metal stripping/plating, such as rate and capacity, affect the functioning of surface patterning, which entails future research attention.

SEI is a decisive factor in tuning interfacial electrodeposition since metal ions need to penetrate an SEI layer and nucleate beneath it.^21^ The SEI layer spontaneously formed in liquid electrolytes is a heterogeneous mixture of insoluble and partially soluble components, leading to uneven ion diffusion through the SEI and accelerating dendrite formation during plating.^22^ In comparison, artificial coatings can be applied onto metal anode surfaces prior to battery assembly, allowing for flexible design of the structure and property of the coating layer. The artificial coating strategy has seen an increase in popularity, including organic or inorganic layers by blade coating, alloying layers by physical/chemical vapor deposition, and chemically grown layers, to optimize metal plating in MBs.^23^ The artificial coatings function as a homogeneous SEI layer to regulate ion transport or participate in generating a uniform SEI layer to realize dendrite-free plating. However, the large-scale preparation of artificial coatings and their compatibility with the processing step are insufficiently studied. For instance, a well-designed artificial SEI layer may be damaged during the subsequent electrode processing (e.g., cutting into shapes).^24^ An artificial coating may not heal itself upon disruption, and hence long-term stability is a stringent requirement. An ideal artificial coating should be rigid and robust to prevent dendrite penetration and at the same time, elastic and flexible enough to withstand volumetric change of the metal anode during repeated cycles.

The three post-treatment strategies discussed in this section facilitate obtaining uniform surface properties and achieving homogeneous electrodeposition of metals (Figure 3c). Up to now, these strategies have only been proven effective in small batches of manually produced metal discs in laboratories. Scalability, repeatability, cost-effectiveness, and compatibility with the battery production process should be taken into consideration when designing new post-treatment strategies.

## 5 Summary and Outlook

Manufacturing of metal anodes from raw materials involves a series of essential steps which need to consider the properties of the metal itself and the compatibility between steps. Establishing a standard pretreatment procedure for a metal ingot can provide a favorable pristine state for efficient processing subsequently. Regarding metal anode processing, roll-press is the most common approach, with which suitable lubricants are the key to suppressing side reactions (oxidation, work hardening) and achieving a desirable thickness of the metal anode. The bottom-up electrodeposition and slurry coating processes are favorable to fabricate complex geometries while processing parameters (e.g., temperature, deposition rate, etc.) need to be optimized to realize desirable morphologies and uniformities. Appropriate post-treatment strategies rectify the diverse imperfections formed in the processing step and enhance the electrochemical performance of the metal anodes. Particularly, improper operations or missing steps can cause issues with the subsequent manufacturing steps. Care needs to be taken during the manufacturing of metal anodes and beyond:

(i) Contaminants may be present in commercially available metal sheets/foils, but they are rarely noted and studied. They can be impurities from raw materials, lubricants oil, dust, or oxides, from processing, packaging, shipping, and storage. They can cause adverse effects on the manufacturing process and electrochemical performance.
(ii) Processing sequence can affect the properties of the final anodes. In a typical example, cutting a metal may introduce fresh edges, which could show many different properties to the post-treatment anode, nullifying the benefits obtained from previous optimization efforts.
(iii) Besides the performance of the anodes, the manufacturing strategies during pretreatment, processing, and post-treatment should be holistically considered in terms of manufacturability, scalability, and cost-effectiveness.
(iv) Considering the highly reactive nature of Li, Na, K, and Ca metals, safety issues during transportation, storage, and manufacturing require a thorough plan and careful execution. This is particularly crucial for large-scale manufacturing.

**Figure 4:**
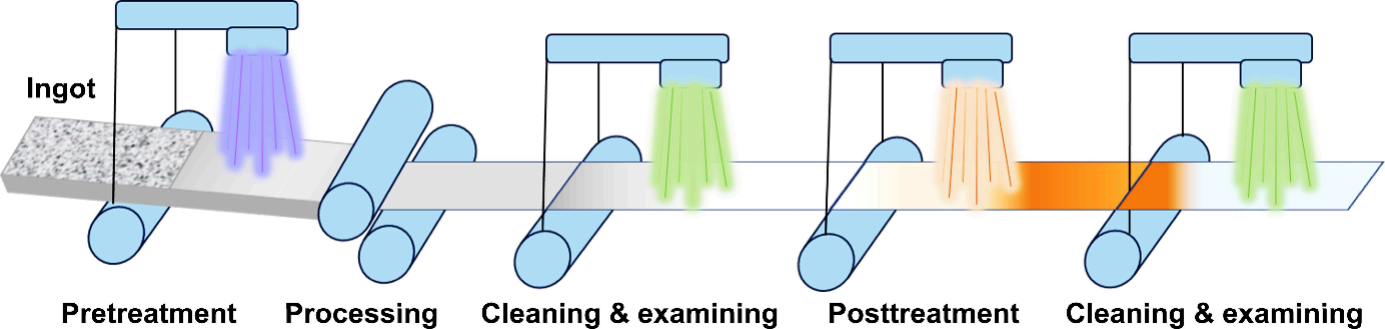
Schematic illustration of a scalable and continuous manufacturing process for metal anodes.

A continuous manufacturing process for scalable production of metal anodes is proposed in Figure 4. An ideal outcome would be that a good pretreatment of raw material is conducive to achieving high-quality processing with little or no imperfections, which can in return ensure an effective post-treatment. Also, it is essential to implement proper, and even better, automated cleaning and quality control measures between the steps. This would significantly improve the production of metal anodes with high quality and low cost. A continuous and automated manufacturing process is effective in avoiding the issues caused by processing sequence and safety hazards during transportation. An integrated manufacturing process for high-quality metal anodes manifests promises and prospects for widespread applications.
